# Fatal occupational accidents in Brazil: A national case-control study using 2023 data

**DOI:** 10.1016/j.clinsp.2025.100798

**Published:** 2025-10-22

**Authors:** Cristine Scattolin Andersen, Victor Alexandre Percinio Gianvecchio, João Silvestre Silva-Junior

**Affiliations:** a Superintendência de Polícia Técnico Científica de São Paulo, Núcleo de Perícias Médico Legais de Santos, Praia Grande, SP, Brazil; b Departamento de Administração e Planejamento da Polícia Civil do Estado de São Paulo, Academia de Polícia Dr. Coriolano Nogueira Cobra (ACADEPOL), São Paulo, SP, Brazil; c Superintendência da Polícia Técnico-Científica de São Paulo, Instituto Médico Legal (IML), São Paulo, SP, Brazil; d Faculdade de Ciências Médicas da Santa Casa de São Paulo, Departamento de Cirurgia, São Paulo, SP, Brazil; e Departamento de Medicina Legal, Bioética, Medicina do Trabalho e Medicina Física e Reabilitação, Faculdade de Medicina da Universidade de São Paulo (FMUSP), São Paulo, SP, Brazil

**Keywords:** Occupational accidents, Mortality, Risk factors, Workforce Vulnerability

## Abstract

**Objective:**

To analyse factors associated with fatal occupational accidents in Brazil in 2023, using data from the Notifiable Diseases Information System (SINAN).

**Methods:**

A quantitative, analytical, retrospective case-control study was conducted with 267,482 occupational accident notifications, of which 2716 (1 %) were fatal. Univariate and multiple logistic regression analyses were performed, with significance set at *p* < 0.05.

**Results:**

Most injured workers were male (77.2 %), non-white (51.9 %), and had at least completed high school (43.9 %). The average age was 36.5-years. About 75.8 % were formally employed, and 41 % had less than one year of job experience. Accidents mainly occurred in the morning (50.5 %) and on the employer’s premises (66.6 %). Logistic regression showed that male workers had a higher risk of fatality (OR=2.59; 95 % CI 2.07–3.24). The risk increased progressively with age, being 3.89-times higher among workers aged 60–65 years than those aged 16–19. Education had a protective effect compared to illiteracy. Workers in the North, Northeast, and Central-West had a 25 % higher risk of death than those in the South and Southeast. Informal work increased fatality odds by 1.77 times, and workers with over 20 years of experience had a 43 % higher risk than those with under one year. Night-time accidents were 1.65 times more lethal than daytime incidents. Race, accident location, type, and body part affected were not significant in multivariate analysis.

**Conclusion:**

Vulnerable groups ‒ men, older workers, the illiterate, informal workers, and those in underdeveloped regions ‒ face higher fatality risks, highlighting the need for targeted policies such as mandatory safety training for informal workers and regional surveillance strategies.

## Highlights

• Male workers showed 2.6-fold higher odds of fatal occupational accidents.
• Informal employment raised fatality risk by 77 % versus formal contracts.
• Night-time accidents were 65 % more lethal than daytime incidents.
• Older age, illiteracy, and poorer regions markedly increased lethality.

## Introduction

In Brazil, the health authority defines an occupational accident as any non-natural injury or violence that occurs in the workplace or while work is being performed.^1^ Events may arise during activities inherent to the worker’s function or while acting on the employer’s behalf (typical accidents), as well as during commuting between home and work (commuting accidents).^1^ A fatal occupational accident is one that leads to death immediately or at any later time, provided the basic cause of death is attributable to the accident.^2^

The International Labour Organization (ILO) estimated that 2.93 million workers died from work-related conditions in 2019 ‒ an increase of more than 12 % compared with 2000.^3^ In Brazil, 410,228 occupational accidents were reported to health authorities in 2023, predominantly involving young, brown-skinned men with low educational attainment.^4^ Among these incidents, 3445 resulted in death and 5284 caused some degree of permanent disability.^4^ External causes rank as the country’s fourth leading cause of death, with work-related fatalities accounting for 3 % of all external-cause deaths.^4^

Beyond the obvious personal, familial, and social losses ‒ whose impact is difficult to quantify ‒ work-related injuries also generate substantial economic costs. Up to 4 % of the national gross domestic product is spent on the direct and indirect consequences of occupational injuries and illnesses.^5^^,^^6^ The scale of this economic and social burden becomes clearer when considering that, according to the ILO, occupational accidents are entirely preventable; their persistence therefore reflects negligence and social injustice.^3^^,^^7^ Effective prevention depends on detailed knowledge of the factors that precipitate such events and of the magnitude of the associated risks.^6^^,^^8^

Occupational accidents are not single-cause phenomena but complex events arising from multiple interacting factors.^9^^,^^10^ Only by thoroughly understanding these factors can public- or private-sector policies ‒ whether individual or collective ‒ be devised to neutralise or, where possible, eliminate the determinants that generate, facilitate or aggravate work-related harm.^6^^,^^8^

Previous studies in Brazil illustrate these dynamics. A 2012 investigation in the State of Paraná documented that almost all fatal occupational accidents between 2006 and 2010 involved men (92 %), mainly aged 19–30 years, with 76 % working under formal contracts; typical accidents accounted for 52 % of cases.^8^ Similarly, a 2015 study in Palmas, State of Tocantins, using DATASUS data found that fatal accidents from 2007 to 2015 occurred chiefly in men (93.8 %), brown-skinned individuals (54.4 %), and those aged 30–49 years (46.5 %), with less than eight years of schooling (52.1 %), predominantly in construction or agriculture (each 26.5 %).^11^ In the State of Bahia, analysis of Mortality Information System (SIM) records from 2000 to 2019 showed that 51 % of work-related deaths occurred among 30- to 49-year-olds; nearly 80 % were typical accidents, yielding an average of 3238 potential years of life lost. Brown-skinned individuals sustained 40.7 potential years of life lost per 100,000 inhabitants, versus 22.6 years among whites.^12^ A 1997 Brazilian study of the metallurgical sector linked low educational level and low wages to increased fatality risk; marital status, neuropsychiatric history, physical debility, and prior injuries were not significant.^13^

By law, occupational accidents are notifiable conditions in Brazil.^14^^,^^15^ All physicians and healthcare facilities ‒ public or private ‒ must report suspected or confirmed cases of notifiable conditions, including work-related injuries, regardless of the worker’s employment status; incidents involving informal workers must also be reported.^1^^,^^16^ Expanding this knowledge base can guide targeted prevention. Accordingly, the present study investigates risk factors associated with fatal occupational accidents in Brazil.

## Methods

This was a quantitative, analytical, retrospective case-control study based on secondary data available from DATASUS, derived from occupational accident notifications in Brazil. These records originate from the compulsory notification forms within the National Notifiable Diseases Information System (SINAN). As the study relied exclusively on publicly available, aggregated, and anonymized secondary data from official information systems, and in accordance with Resolution No. 510/2016 of the Brazilian National Health Council (CNS)—which waives the requirement for Research Ethics Committee review for studies using publicly accessible information with no possibility of identifying individual subjects—this research was exempt from submission to and approval by an Ethics Committee. All notifications from 2023 were included, as this was the most recent year with complete data on the platform.^4^ Statistical analyses were conducted using Jamovi software.

The year 2023 was selected due to a regulatory change issued by Brazil’s Ministry of Health, which expanded the scope of mandatory reporting. Previously, only “severe occupational accidents” were required to be reported. As of March 1st, 2023, under Ordinance GM/MS n° 217, notification of all occupational accidents became mandatory.^15^ To ensure comparability and avoid bias, the study included only notifications from 2023.

Data were collected through the DATASUS portal by following the path: Acesso à Informação > Serviços > Transferência/Download de Arquivos > SINAN > Dados > ACGR - Acidente de Trabalho > 2023. All records classified under ICD-10 chapters S00–T98 (injuries, poisoning, and other consequences of external causes) were included.

The dependent variable was “occupational accident,” with the case group comprising notifications classified as “fatal occupational accidents,” while notifications of “non-fatal occupational accidents” were considered the control group. It is acknowledged that the control group (non-fatal accidents) may not adequately reflect the occupational distribution of the workforce, introducing potential selection bias. However, the absence of information on occupation or economic activity sector precluded matching for these variables.

Independent variables included: age group, sex, race (white/non-white), education level, region of residence, employment type (formal/informal), time in job, accident location, work shift, accident type (typical or commuting), and affected body part.

Descriptive analysis (means, standard deviations, ranges, proportions) was followed by univariate logistic regression. Variables with *p* < 0.20 were included in the multiple logistic regression model using a stepwise procedure, with automatic inclusion and removal of independent variables based on the statistical significance observed in the univariate analysis.

Reference categories were those with the lowest odds of fatality. Variables with *p* < 0.05 in the final model were considered statistically significant. Variables initially significant in univariate analysis were retained even if they lost significance at later stages, to improve model fit. To assess potential multicollinearity among independent variables, variance inflation factors (VIFs) were calculated.

## Results

In 2023, a total of 267,482 occupational accidents resulting from trauma or other external causes were reported to SINAN. Of these, 82.8 % were classified as typical occupational accidents and 17.2 % as commuting accidents. The mean age at the time of the accident was 36.5-years (standard deviation: 12.1). Most injured individuals were male (77.2 %), non-white (51.9 %), had completed at least high school or higher education (43.9 %), and lived in Brazil’s Southeast region (38.7 %). The majority of reports referred to accidents among formally employed workers (75.8 %), and 41.0 % of accidents involved individuals with less than one year of job experience. Most incidents occurred in the morning (50.5 %) and on the employer’s premises (66.6 %). The upper limbs were the most frequently affected body part (49.3 %). Only 2716 cases (1 %) were classified as fatal occupational accidents.

Table 1 presents the full descriptive analysis.

**Table 1:** Description of individual and occupational characteristics of workers involved in occupational accidents in Brazil, 2023, according to SINAN notifications (*N* = 275,365).

Variable	N ( %)	Fatal *N* = 2716	Non-fatal *N* = 272,649
**Age group (*N*=267,482)**			
16–19 years	11,462 (4.3 %)	53 (0.0 %)	11,409 (4.3 %)
20–29 years	82,328 (30.8 %)	500 (0.2 %)	81,828 (30.6 %)
30–39 years	68,853 (25.7 %)	572 (0.2 %)	68,281 (25.5 %)
40–49 years	57,920 (21.7 %)	653 (0.2 %)	57,267 (21.4 %)
50–59 years	36,539 (13.7 %)	487 (0.2 %)	36,052 (13.5 %)
60–65 years	10,380 (3.9 %)	249 (0.1 %)	10,131 (3.8 %)
**Sex (*N*=275,353)**			
Male	212,625 (77.2 %)	2490 (0.9 %)	210,135 (76.3 %)
Female	62,728 (22.8 %)	226 (0.1 %)	62,502 (22.7 %)
**Race (*N*=271,618)**			
White	130,567 (48.1 %)	1200 (0.4 %)	129,367 (47.6 %)
Non-white	141,051 (51.9 %)	1494 (0.6 %)	139,557 (51.4 %)
**Education level (*N*=258,824)**			
Illiterate	55,498 (21.4 %)	677 (0.3 %)	54,821 (21.2 %)
Incomplete elementary	43,487 (16.6 %)	585 (0.2 %)	42,902 (16.6 %)
Complete elementary	46,205 (17.9 %)	466 (0.2 %)	45,739 (17.7 %)
High school/college or more	113,634 (43.9 %)	808 (0.3 %)	112,826 (43.6 %)
**Region of residence (*N*=275,322)**			
North	16,889 (6.1 %)	253 (0.1 %)	16,636 (6.0 %)
Northeast	28,932 (10.5 %)	386 (0.1 %)	28,546 (10.4 %)
Southeast	106,499 (38.7 %)	958 (0.3 %)	105,541 (38.3 %)
South	97,107 (35.3 %)	777 (0.3 %)	96,330 (35.0 %)
Central-West	25,895 (9.4 %)	342 (0.1 %)	25,553 (9.3 %)
**Employment status (*N*=272,388)**			
Formal	206,544 (75.8 %)	1550 (0.6 %)	204,994 (75.3 %)
Informal	65,844 (25.2 %)	1073 (0.4 %)	64,771 (23.8 %)
**Work experience (*N*=191,709)**			
< 1 year	78,649 (41.0 %)	377 (0.2 %)	78,272 (40.8 %)
1–5 years	61,019 (31.8 %)	364 (0.2 %)	60,655 (31.6 %)
6–9 years	20,071 (10.5 %)	115 (0.1 %)	19,956 (10.4 %)
10–14 years	13,185 (6.9 %)	113 (0.1 %)	13,072 (6.8 %)
15–19 years	6447 (3.4 %)	67 (0.0 %)	6380 (3.3 %)
20–35 years	12,338 (6.4 %)	186 (0.1 %)	12,152 (6.3 %)
**Accident location (*N*=263,323)**			
Employer's premises	175,281 (66.6 %)	858 (0.3 %)	174,423 (66.2 %)
Public roads	57,457 (21.8 %)	1247 (0.5 %)	56,210 (21.3 %)
Third-party premises	19,686 (7.5 %)	334 (0.1 %)	19,352 (7.3 %)
Worker’s home	10,899 (4.1 %)	119 (0.0 %)	10,780 (4.1 %)
**Time of accident (*N*=242,638)**			
Morning	122,644 (50.5 %)	943 (0.4 %)	121,701 (50.2 %)
Afternoon	95,841 (39.5 %)	893 (0.4 %)	94,948 (39.1 %)
Night	24,153 (10.0 %)	315 (0.1 %)	23,838 (9.8 %)
**Type of accident (*N*=269,889)**			
Typical	223,558 (82.8 %)	1790 (0.7 %)	221,768 (82.2 %)
Commuting	46,331 (17.2 %)	794 (0.3 %)	45,537 (16.9 %)
**Body part affected (*N*=264,753)**			
Head	45,252 (17.1 %)	1114 (0.4 %)	44,138 (16.7 %)
Trunk	11,688 (4.4 %)	363 (0.1 %)	11,325 ((4.3 %)
Upper limbs	130,618 (49.3 %)	83 (0.0 %)	130,535 (49.3 %)
Lower limbs	72,007 (27.2 %)	81 (0.0 %)	71,926 (27.2 %)
Whole body	5188 (2.0 %)	932 (0.4 %)	4256 (1.6 %)

Univariate logistic regression analysis revealed statistical significance for all independent variables in relation to the outcome (fatal occupational accident), leading to their inclusion in the multiple model. However, the variables race, accident location, accident type, and affected body part lost statistical significance and were retained in the final model solely for adjustment purposes.

As shown in Table 2, sociodemographic factors such as sex, age group, education level, and region of residence were significantly associated with the outcome. Male workers had nearly three times the odds of fatal injury compared to females (OR=2.59; 95 % CI 2.07–3.24). A positive age gradient was observed: workers aged 60–65 had a 3.89-fold higher risk of death compared to those aged 16–19.

**Table 2:** Univariate and multiple logistic regression analysis of factors associated with fatal occupational accidents notified in SINAN, Brazil, 2023 (*N* = 267,482).

Variable	Univariate OR (95 % CI)	p-value	Multiple OR (95 % CI)	p-value
Age group				
16–19 y	1.00 (ref)	—	1.00 (ref)	—
20–29 y	1.32 (0.99–1.74)	0.058	1.07 (0.71–1.60)	0.800
30–39 y	1.80 (1.36–2.39)	<0.001	1.52 (1.02–2.29)	0.040
40–49 y	2.45 (1.85–3.25)	<0.001	1.91 (1.28–2.88)	0.002
50–59 y	2.91 (2.19–3.86)	<0.001	2.31 (1.52–3.51)	<0.001
60–65 y	5.29 (3.92–7.12)	<0.001	3.89 (2.48–6.09)	<0.001
Sex				
Female	1.00 (ref)	—	1.00 (ref)	—
Male	3.28 (2.86–3.76)	<0.001	2.59 (2.07–3.24)	<0.001
Education				
Illiterate	1.00 (ref)	—	1.00 (ref)	—
Incomplete elementary	1.10 (0.99–1.23)	0.081	0.71 (0.58–0.87)	<0.001
Complete elementary	0.82 (0.73–0.93)	0.001	0.74 (0.60–0.91)	0.004
High school or more	0.58 (0.52–0.64)	<0.001	0.74 (0.62–0.89)	0.001
Region				
South/Southeast	1.00 (ref)	—	1.00 (ref)	—
North/Northeast/Central-West	1.61 (1.49–1.75)	<0.001	1.25 (1.06–1.47)	0.006
Employment status				
Formal	1.00 (ref)	—	1.00 (ref)	—
Informal	2.19 (2.03–2.37)	<0.001	1.77 (1.54–2.04)	<0.001
Work experience				
< 1 year	1.00 (ref)	—	1.00 (ref)	—
1–5 years	1.25 (1.08–1.44)	0.003	1.09 (0.93–1.28)	0.300
6–9 years	1.20 (0.97–1.47)	0.093	0.92 (0.72–1.16)	0.500
10–14 years	1.79 (1.45–2.22)	<0.001	1.18 (0.92–1.50)	0.200
15–19 years	2.18 (1.68–2.83)	<0.001	1.26 (0.93–1.72)	0.100
20–35 years	3.18 (2.66–3.79)	<0.001	1.43 (1.14–1.79)	0.002
Shift				
Daytime	1.00 (ref)	—	1.00 (ref)	—
Night	1.56 (1.38–1.76)	<0.001	1.65 (1.37–1.99)	<0.001

Education appeared to be a protective factor, with all educational levels associated with a lower fatality risk than illiteracy. Living in the North, Northeast, or Central-West regions was associated with a 25 % higher chance of fatality compared to living in the South or Southeast.

Work-related conditions also showed significant associations: informal employment increased the odds of death by 1.77 times, while workers with more than 20 years of experience had a 43 % higher fatality risk compared to those with less than one year. Accidents occurring at night were 1.65 times more likely to be fatal than those occurring during the day. Table 2 presents the results of the multiple logistic analysis.

All variables included in the final regression model had VIF values < 2, indicating that multicollinearity was unlikely to have influenced the estimated associations. The model demonstrated acceptable fit, with AIC (11,780) and BIC (11,860) values supporting its parsimony. The pseudo-R² (0.0373) indicated that the independent variables explained only a small proportion of the variability in fatal occupational accidents, which is consistent with expectations for complex and multifactorial outcomes assessed using secondary surveillance data.

## Discussion

This study, based on 2023 SINAN notifications of occupational accidents in Brazil, identified an increased risk of fatality among workers who were male, aged over 20-years, illiterate, residing in the North, Northeast, or Central-West regions, with over 20-years of work experience, employed in the informal labor market, and whose accidents occurred during the night shift.

Fatal occupational accidents were more frequent among male workers, corroborating previous literature.^8^^,^^11^ This pattern is likely due to the concentration of men in high-risk sectors such as agriculture, industry, and construction.^11^ These sectors also commonly employ workers with low educational attainment, who are more vulnerable to occupational injuries. In contrast, higher educational levels are generally associated with less hazardous occupations,^9^^,^^17^ aligning with the authors’ finding that education appears to be a protective factor against fatal occupational accidents. It should be noted, however, that the SINAN database does not include key variables such as productive sector, type of occupation, or history of occupational safety training. The absence of such information significantly limits the ability to control for confounding variables, which may distort the interpretation of the findings. This bias may, for example, contribute to spurious or overestimated associations, such as the apparent protection conferred by higher education levels, which may in fact reflect the placement of these individuals in sectors with lower occupational risk exposure.

Age was statistically associated with accident fatality in this study. Although the average age of injured workers was 36.5-years, the fatality risk increased with age, particularly after age 30. However, this association is not consistent across the literature. A review of studies from the U.S., Sweden, and Canada reported that 56 % of investigations identified workers under 25 as the most accident-prone, 17 % found the opposite, and 27 % found no significant differences across age groups.^18^^,^^19^

Interestingly, although 41 % of the accidents occurred among workers with less than one year of experience, the highest fatality risk was among those with more than 20-years of experience. While lack of experience may be linked to riskier behavior and higher accident frequency,^9^^,^^20^ more experienced workers may face fewer accidents but of greater severity ‒ potentially due to operational failures rather than inattention or disregard for safety protocols.^9^

Informal employment was another risk factor, consistent with previous findings showing that informal workers often have little or no education and limited access to occupational health and safety services.^9^^,^^21^^,^^22^ Such precarious conditions may lead to increased exposure to hazards and reduced adherence to safety practices, contributing to higher fatality rates. The present findings showed that informal workers had a 1.77-fold greater risk of fatal occupational injury than their formally employed counterparts.

Night work also emerged as a risk factor, echoing current literature. Night shifts are known to impair sleep quality, leading to physical and mental fatigue, cognitive and emotional impairments, reduced immunity, and increased risk of metabolic disorders.^23^ A German study found that sleep deprivation among workers caused fatigue and low energy, which in turn led to inattentiveness, unsafe behaviours, and a higher likelihood of severe accidents.^24^^,^^25^ It should also be noted that, in addition to fatigue, the higher lethality observed during night shifts may be related to the reduced availability of emergency teams, medical support services, and institutional supervision at night. Studies indicate that the response to critical events tends to be slower and less effective during this period, which can worsen outcomes, particularly in contexts with higher occupational risk.^26^^,^^27^

This study also found that the highest rates of reported occupational accidents occurred in the South (35.3 %) and Southeast (38.7 %) regions of Brazil. However, the North, Northeast, and Central-West regions showed an increased risk of fatal occupational accidents. On this topic, there is no consensus in the literature. In the study by Malta et al., which analyzed occupational accidents from 2013 to 2019, the worst outcomes were observed in the North, Northeast, and Central-West states, possibly due to the predominantly rural economic activities in these areas, which are more frequently associated with accidents involving animal handling, machinery, sharp tools, and contaminated materials.^17^ It is noteworthy that the North, Northeast, and Central-West regions concentrate high-risk activities such as agriculture, livestock farming, and extractivism, and have lower coverage of emergency services and labor inspection, which may contribute to worse outcomes.^17^ Another study, published in 2021, conducted a temporal analysis of occupational accident mortality between 2006 and 2015 and found that the South region had the highest mortality rates in the country, a result that the authors associated with the greater efficiency of the region’s reporting system.^9^

The quality of Brazil’s compulsory notification system, however, represents the main limitation of this study. Although notification is mandatory and its omission constitutes a criminal offense^28^ and a potential breach of professional ethics,^29^ there is a well-documented and substantial underreporting of work-related injuries and illnesses in the country. Studies estimate that only one in every ten diagnosed work-related conditions is effectively reported to the authorities,^5^^,^^7^ creating a substantial gap between official data and the true epidemiological situation. Several factors contribute to this underreporting. First, there are structural barriers within health services and information systems, which often lack integrated workflows and adequate training for professionals to identify and record work-related cases. Additionally, fear of retaliation by employers and the precarious nature of labor relations — particularly in informal or outsourced contexts—lead workers themselves to refrain from reporting incidents, fearing job loss or stigmatization.^5^^,^^7^ Another critical factor is the difficulty in establishing a causal link between the event (including death) and occupational activity. Many deaths that are in fact related to hazardous or unhealthy working conditions are recorded under generic external causes, such as traffic accidents, homicides, or ill-defined events. This contributes to the statistical invisibility of occupational accidents and diseases, reducing their priority in public health and occupational safety policies.^9^

Underreporting directly affects the formulation and implementation of public policies, as it undermines the identification of high-risk sectors, the allocation of resources for prevention and assistance, and the accountability of employers. Furthermore, it distorts estimates of the economic costs associated with occupational accidents, such as sick leave, early retirement, and productivity loss.^9^ In light of this scenario, it is reasonable to assume that the true magnitude of the problem is significantly greater than what is reflected in official databases—possibly exceeding current figures by a factor of ten, as previously estimated by other studies.^5^^,^^7^ Bridging this gap requires strengthening worker health surveillance strategies, fostering intersectoral integration of information systems, and promoting a culture of active and responsible reporting.

## Conclusion

This study identified several risk factors associated with the fatality of occupational accidents in Brazil: being male, older age, lack of formal education, residing in the North, Northeast, or Central-West regions, working in the informal labor market, having over 20 years of job experience, and experiencing an accident during the night shift. Understanding these factors enables the development of specific prevention actions at organizational and governmental levels, aimed at reducing the incidence, severity, and lethality of occupational accidents.

Given that this study relied on secondary data from national records of notifiable conditions, a major limitation lies in the issue of underreporting and the variable quality of the data. It is crucial for Brazil to strengthen the training and engagement of healthcare professionals to improve the quality and completeness of the reporting system. Such improvements would support a more accurate understanding of the country’s health conditions and enable better-informed decision-making by professionals and policymakers.

There remains a clear need for further research on the risk factors for fatal occupational accidents, particularly studies based on primary data to mitigate the limitations posed by underreporting in national surveillance systems. In addition, promoting research with stronger causal inference ‒ such as longitudinal or experimental designs ‒ would greatly enhance the evidence base for targeted and effective interventions.

## Declaration of competing interest

The authors declare no conflicts of interest.
